# Risky sexual practices and associated factors among taxi drivers in the Finoteselam town, northwest Ethiopia, 2023: a community-based cross-sectional study

**DOI:** 10.3389/frph.2024.1436615

**Published:** 2024-11-27

**Authors:** Melaku Laikemariam, Yetwale Fetene

**Affiliations:** ^1^ Department of Midwifery, College of Health Sciences, Debre Markos University, Debre Markos, Ethiopia; ^2^ Department of Reproductive Health, Finoteselam Health Center, Finoteselam, Ethiopia

**Keywords:** risky sexual practice, sexual behaviour, adverse sexual practice, taxi drivers, Ethiopia

## Abstract

**Background:**

Risky sexual practices among taxi drivers pose a significant threat to public health, contributing to the spread of sexually transmitted infections (STIs) and HIV/AIDS. The nature of their profession often exposes taxi drivers to factors that increase their vulnerability to engaging in risky sexual practices. Although research on sexual health in general is readily available, studies specifically focused on this population are limited. Understanding the contributing factors behind risky sexual practices among taxi drivers is crucial to developing targeted interventions that address their unique needs and vulnerabilities.

**Objectives:**

To assess the prevalence and associated factors of risky sexual practices among taxi drivers in Finoteselam town, northwest Ethiopia, 2023.

**Methods:**

A cross-sectional study was conducted among 359 taxi drivers in the Finoteselam town. Data were collected using pre-tested questionnaires and analyzed using SPSS. Bivariate and multivariable analyses were performed to identify factors associated with risky sexual practices. In the bivariate analysis, a *p*-value ≤ 0.25 at 95% CI was used to consider the variables in the multivariate analysis. Finally, statistical significance was declared with a *p*-value of less than 0.05 with a 95% CI.

**Results:**

The prevalence of risky sexual practices among taxi drivers was 32.9 (95% CI: 28.01, 38.7). Living alone (AOR = 3.47, 95% CI: 1.86, 6.48), taxi ownership (AOR = 2.08, 95% CI: 1.01, 4.25), neglecting the discussion of the SRH issue (AOR = 2.05, 95% CI: 1.08, 4.00), substance use (AOR = 1.56, 95% CI: 1.04, 2.09), attending night clubs (AOR = 6.04, 95% CI: 1.97, 18.55) and watching pornographic materials (AOR = 4.44, 95% CI: 2.14, 9.19) were significantly associated with risky sexual practices.

**Conclusions and recommendation:**

This study revealed a high prevalence of risky sexual practices among taxi drivers in Finoteselam town. Therefore, a comprehensive approach involving different stakeholders is required for reducing risky sexual practices. Information dissemination, awareness creation (about risks of attending nightclubs, substance use, and watching pornography), and encouraging knowledge sharing about sexual health are some of the interventions required to reduce risky sexual practices among taxi drivers.

## Introduction

Risky sexual practices are any form of sexual activity that contributes to the spread of STIs and HIV/AIDS (1) and is commonly experienced in the taxi industry (2). Sexual practices such as premarital sex, multiple sexual partners, unprotected sex, and sex with prostitutes are commonly experienced risky practices by taxi drivers (3, 4). Risky sexual practices result in infections believed to be one of the main causes of preventable mortality in developing nations, including Ethiopia (5). Due to their work habits, taxi drivers did not get access to information and support about their sexual health, and therefore, participation in risky sexual practice was significantly higher, and the odds of risky sexual practice in nations without access to high-quality reproductive health care are higher (6–8).

Due to risky sexual conduct, 14,000 new HIV infections occur worldwide every day; more than 95% of these infections occur in sub-Saharan Africa (9–11). The proportion of sex before marriage (premarital sex) (12) and non-communication about reproductive health issues is significantly higher in developing nations, including Ethiopia (13). Neglecting sexual and reproductive health concerns results in serious reproductive and sexual health problems (11, 13–16).

In many developing countries, taxi drivers were recognised as vulnerable community members for risky sexual practices (17–20); furthermore, the odds of risky sexual practice among taxi drivers was significantly higher (21). As a result of risky sexual practices, the magnitude of STDs and HIV/AIDS among taxi drivers and their respective assistants increased (22). The habit of condom use among taxi drivers was significantly low (23). Studies conducted in Bangladesh showed that risky sexual practices such as unprotected sex, inconsistent condom use, premarital sex, multiple sexual partners among taxi drivers were significantly higher (24–26). According to the study, strong family connection was identified as a protective factor against risky sexual practices (27).

Substance use and alcohol consumption had far-reaching implications for risky sexual practices, which have been demonstrated through studies (25, 26, 28–30). The habit of discussing sexual and reproductive health issues with your family improved the taxi driver sexual practices and reproductive life (31–33), but pressure from peers increased the likelihood of engaging in risky sexual practices (31). As a result of this, the promotion of good communication habits about SRH issues promotes the sexual and reproductive health of people (33). The absence of parental control over individual sexual and reproductive practices promotes the practices of risky sexual acts (34), but a study conducted in Ghana found that family communication did not affect the individuals sexual practices (35). Absence of parental control over individual sexual and reproductive issues improves risky sexual practices (33).

The odds of risky sexual practices among those who had the habit of watching audio-visual pornographic audiovisual material were significantly higher (36). Studies conducted in the USA have shown that exposure to sexually explicit websites was significantly linked to risky sexual practices (37) and pornographic materials change their perceptions of themselves and their sexual practices (38). Another study showed that the habit of watching pornographic materials (39) and the frequent engagement in watching of pornographic materials (40) promote risky sexual practices among the individuals. Taxi drivers who had the habit of attending nightclubs and bars engaged in risky sexual practices under the influence of illegal substances and excessive alcohol consumption (41).

Although there is a strong effort to improve reproductive and sexual health, the incidence of STI and HIV/AIDS is a result of risky sexual practices (2, 26, 40). Although the scientific evidence is a baseline to develop interventional strategies and activities to reduce sexual health problems, there is limited evidence of risky sexual practice in the study area. Long working hours, irregular schedules, and contact with a wide range of passengers make taxi drivers more vulnerable to risky sexual practices, so the conduct of this scientific evidence is important for reducing risky sexual practices induced sexual and reproductive health problems among taxi drivers. The current study is significantly important for providing baseline information for health policy makers and reproductive health experts to prioritise the study population. The findings of the research provide a baseline for the development of awareness creation interventions for taxi drivers. Therefore, the current study aimed to assess the prevalence and associated factors of risky sexual practices among taxi drivers in Finoteselam town, northwest Ethiopia, in 2023.

## Methods and materials

### Study design, study period, and study setting

A community-based cross-sectional study was conducted in the Finoteselam town, northwest Ethiopia, from September 1 to October 30, 2023. The study area is located in the West Gojjam district of the Amhara region, and the finote selam town serves as the west Gojjam zonal administrative site. The finote Selam town is located 387 km from the Addis Ababa and 176 km from administrative town of the region called Bahir Dar town. The city administration has four urban and two rural Kebeles. According to the 2007 national census conducted by the Central Statistical Agency of Ethiopia (CSA), this town has a total population of 25,913 people, 13,035 of whom are men and 12,878 of whom are women. According to the report of the town transport administrative department, there are more than 1,000 automobile taxis with their respective drivers available in the town. Despite the presence of a large number of taxis, only 798 taxi drivers were serving the community by joining formal taxi associations.

### Population

All taxi drivers in the town were a source populations, while those randomly selected taxi drivers from similar settings were study populations.

### Eligibility criteria

All taxi drivers who agreed to participate were included in the study, while those who suffered of sever medical illness and were not member of taxi drivers association during the study period were excluded from the study.

### Sample size determination

The required sample size was determined with the single population proportion formula using the *p*-value of risky sexual practices (30.6%) among transport workers (21). The sample size was determined using a 5% margin of error and a 5% level of significance (two-sided). The sample size was calculated as follows:

$$\begin{array}{rl} & n\;=\frac{{\left(Z\alpha /2\right)}^{2}\;p(1-p)}{d^{2}}\\ & n\;=\;\frac{{\left(1.96\right)}^{2}\;\times \;0.306\;(1-0.306)}{{\left(0.05\right)}^{2}}\;=\;326.19 \end{array}$$

The final sample size was 375.12∼376 with a 15% non-response rate.

### Sampling procedure

**Figure 1:**
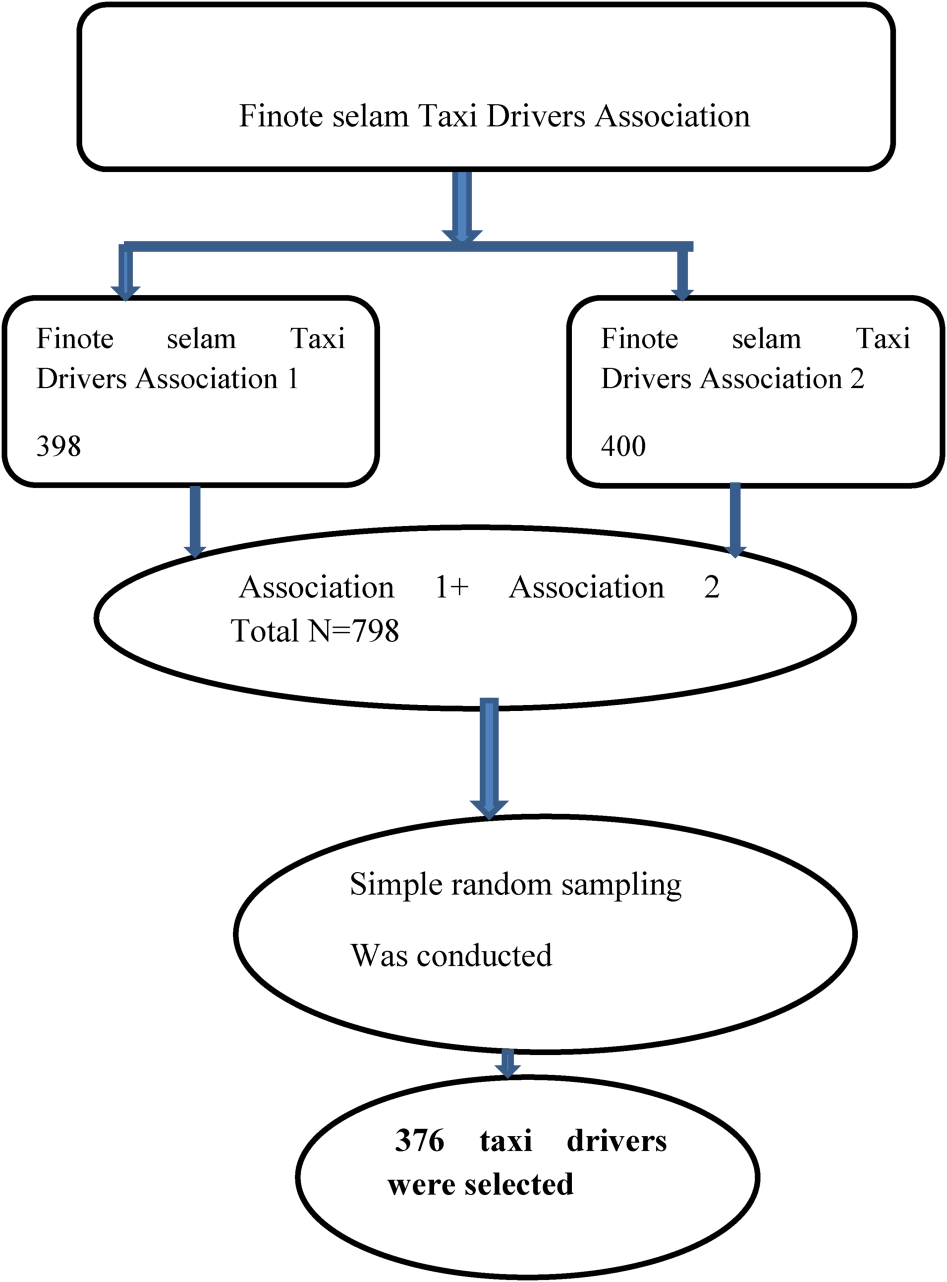
Schematic representation of the sampling procedure to select taxi drivers in the Finoteselam town, northwest Ethiopia, 2023.

There are two taxi associations with a total membership of 798 taxi drivers in the town. Each taxi side number or code was obtained from the taxi association logbook after merging of the two taxi associations. By taking the taxi side number or assigned code number as the sampling frame, a simple random sampling method was used to draw 376 taxi drivers (see Figure 1).

### Variables

The dependent variable of the study was risky sexual practices, whereas the independent variables were sociodemographic characteristics (age, marital status, educational level, living status, monthly income, residency, religion, experience driving and taxi ownership), lifestyle habits [substance use (alcohol, chat, and cigarettes), pornographic view, attending night clubs, having intimate friends who started sexual contact] and sexual behaviour (ever started sex, number of sexual partners, habit of condom use).

### Operational and term definitions

#### Risky sexual practices

Taxi drivers who have one or more of the following sexual practices: premarital sex, multiple sexual partners, unprotected sexual practices, or inconsistent use during sexual acts (21, 42–44).

#### Substance use

Alcohol consumption, khat chawing, smoking, use of shisha and related substances that alter the individuals conscious judgment of the individual (45).

#### Premarital sex

A practice of performing sexual activity before marriage (46).

#### Inconsistent condom use

Interrupted, incorrect, and occasional use of a condom during episodes of sexual activity with at least one nonmarried and extra-sexual partner.

#### Multiple sexual partners

Taxi who had ever had an additional sexual partner at least once in their lifetime until the study period.

#### Unprotected sexual practice

Act of performing sexual contact with nonmarried or extra sexual partners without the use of a condom.

### Data collection tools and technique

Six data collectors collected data by using a pretested structured questionnaire and a checklist prepared in the local language. Two health officers supervised the data collection. The questionnaire was adapted from related previous studies in which their validity was tested (21, 43, 45). Data collectors approached the study participants on the time waiting for their respective round at taxi terminals (fermata). The data collectors approached the taxi drivers using randomly selected respective taxi side number or the town code number of the taxi at the taxi terminals (fermata) of the town. Questionnaires prepared in the local language were provided to each taxi drivers to complete them.

### Data quality assurance

Two weeks before the actual study, a pretest was conducted among 5% (19 taxi drivers) of the total of taxi drivers in Burie town. After the pretest, the necessary amendments were made to the questionnaires. Confusing and unclear questions were checked and edited accordingly prior to actual data collection. Regular check-ups for completeness and consistency of the data were performed daily. The English version of the questionnaire was translated to Amharic (a local language) and then retranslated back to English by two language experts. Three days of training was provided to data collectors and supervisors. After the data was collected, the data were entered into the epi info version. Furthermore, during data analysis, the data were cleaned and any missing values were carefully handled.

### Data processing and analysis

Data were cleaned to ensure completeness, consistency, the absence of missing values, and appropriate variable coding. After the data was collected and entered in to Epi Info version 7, the collected data were exported to SPSS version 26 for analysis. The sociodemographic characteristics of the taxi drivers were analysed using a descriptive analysis of SPSS. A binary logistic regression was performed to identify factors associated with risky sexual practices. A *p*-value < 0.05 at 95% CI was used in the bivariate (bivariable) analysis to consider variables in the multivariate (multivariable) analysis and a *p*-value < 0.05 at 95% CI was used to declare final significance of the association. A model fit test was conducted using the Hosmer and Lemeshow test, and data presentation techniques such as percentages, frequency distribution tables and figures were used to present the findings.

## Results

### Sociodemographic characteristics

A total of 359 taxi drivers participated and a response rate of 95.48%. Most of the taxi drivers were male (356 (99.2%) males and 3 (0.8%) females). The mean age of the taxi drivers was 27.73 years (SD = ±5.7). The mean age of the taxi drivers was 27.73 years (SD = ±5.7) and 259 (72.1%) were taxi owners. Most of taxi drivers were living with their families (199, 55.4%) (see Table 1**)**.

**Table 1:** Sociodemographic characteristics of taxi drivers in the Finoteselam town, northwest Ethiopia, 2023.

Variables	Categories	Frequency	Percentage (%)
Age	18–24 years	80	22.3%
	25–34 years	209	58.2%
	≥35 years	70	19.5%
Residence	Urban	312	86.9%
	Rural	47	13.1%
Religion	Orthodox	289	80.5%
	Muslims	56	15.6%
	Others^a^	14	3.9%
Marital status	Never married	176	49.1%
	Married	152	42.3%
	Others^b^	31	8.6%
Educational status	Completed 1^ry^ school	27	7.5%
	Completed 2^ry^ school	191	53.2%
	College and above	141	39.3%
Living arrangement	Live alone	160	44.6%
	Live with family	199	55.4%
Taxi ownership	Owner of taxi	259	72.1%
	Non owner of taxi	100	27.9%
Work experience	Less than 5 years	170	47.4%
	Greater or equal to 5 years	189	52.6%
Monthly income	<5,000 ETB	67	18.7%
	≥5,000 ETB	292	81.3%

^a^ Protestant and without religion.

^b^ Divorced and widowed.

### Lifestyle habits and behavioural characteristics of taxi drivers

Most taxi drivers (190, 52.9%) used different types of substances, and the most commonly used substance was alcohol [102, 53.7%]. Even the majority of taxi drivers [273 (76%)] perceived the risk of STDs, 126 (35.1%) of them neglected the discussion of the issue of SRH. Among taxi drivers, 89 (24.8%) and 43 (12%) of them had the habit of watching pornography and attending nightclubs, respectively (see Table 2).

**Table 2:** Lifestyle habits and behavioural characteristics of taxi drivers in finoteselam town, northwest Ethiopia, 2023.

Variables	Categories of variables	Frequency	Percentage (%)
Substance use	Yes	190	52.9%
	No	169	47.1%
Type of substance use^b^	Alcohol	102	53.7%
	Khat	37	19.5%
	Cigarette	29	15.3%
	Others^a^	14	7.4%
	More than 2 types of substance	8	4.2%
Intimate friend who started sex	Yes	162	45.1%
	No	197	54.9%
SRH issue discussion	No	126	35.1%
	Yes	233	64.9%
Watching pornography	Yes	89	24.8%
	No	270	75.2%
Attending nightclub	Yes	43	12%
	No	316	88%
Perceived risk of STDs	No	86	24%
	Yes	273	76%
History of STI/STDs	Yes	26	9.5%
	No	248	90.5%

SRH-, sexual and reproductive health.

^a^ Shisha, cannabis and related illicit drugs.

^b^ More than one choice possible, parents and their sexual partners.

### Sexual practices of taxi drivers in finote selam town

**Figure 2:**
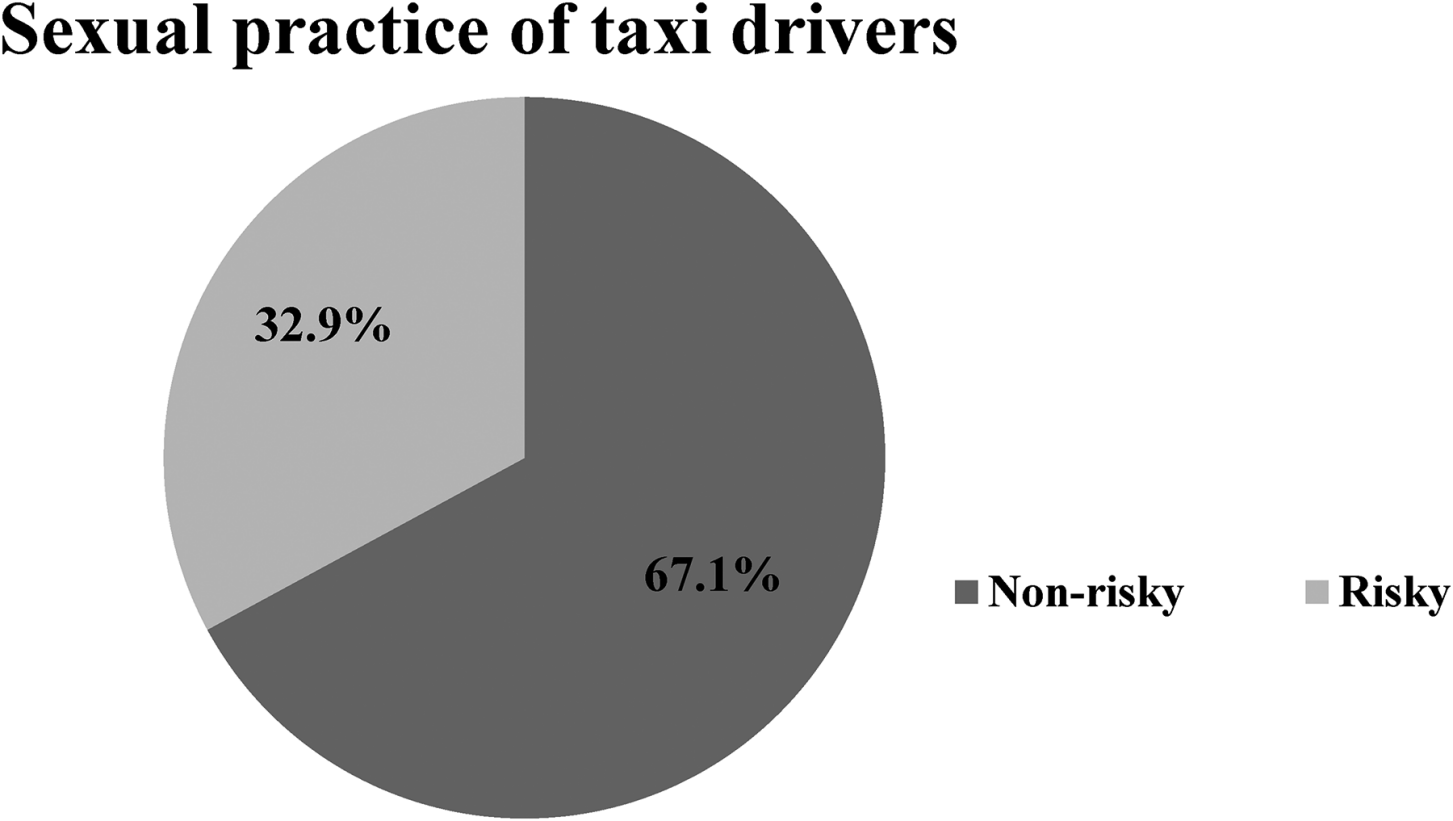
Sexual practice of taxi drivers in the Finoteselam town, northwest Ethiopia, 2023.

The prevalence of risky sexual practices among taxi drivers was 32.9 (95% CI: 28.01, 38.7**).** Among taxi drivers, 105 (38.3%) of them had premarital sex, and 107 (39.1%) had unprotected sexual practices (see Table 3 and Figure 2).

**Table 3:** Sexual practices and characteristics of taxi drivers in the finoteselam town, northwest Ethiopia, 2023.

Variables	Categories	Frequency	Percentage (%)
Ever had sexual contact	Yes	274	76.3%
	No	85	23.7%
Multiple sexual partners	Yes	92	33.6%
	No	182	66.4%
Number of sexual partners	2 partners	44	47.8%
	3 partners	41	44.6%
	>3 partners	7	7.6%
Premarital sex	Yes	105	38.3%
	No	169	61.7%
Use of condom during sex	No	107	39.1%
	Yes	167	60.9%
condom use status	Inconsistently	48	28.7%
	Consistently	119	71.3%
Sexual practice	Risky	118	32.9%
	Non risky	241	67.1%

### Factors associated with risky sexual practices

In the bivariate analysis, characteristics such as age, residence, education status, current living arrangement, status of taxi ownership status, having intimate friends who started sexual contact, previous history of STI, perceived risk of STI and HIV/AIDS, discussion of sexual and reproductive health (SRH) issues with family, habit of substance use, attending night clubs and watching sex-eliciting audio-visual materials were variables associated with risky sexual practices of taxi drivers at *p* ≤ 0.25 at 95% confidence levels.

In the multivariate analysis, current living arrangement, taxi ownership, substance use, discussion of the SRH issue, attending nightclubs and watching pornography were significantly associated with risky sexual practices at *p* values < 0.05 with a 95% confidence level. Accordingly, the likelihood of risky sexual practices among taxi drivers who lived alone was three times greater than among those living with family (AOR = 3.47, 95% CI 1.86, 6.48). Taxi drivers who drove their own taxi had twice the risk of experiencing risky sexual practices (AOR = 2.08, 95% CI: 1.01, 4.25). The odds of risky sexual practices among taxi drivers who neglected to discuss the SRH issue were twofold greater than those who discussed the SRH issues (AOR = 2.05, 95% CI 1.08, 4.00). The odds of risky sexual practices among substance users taxi drivers were 1.5 times greater than among nonusers (AOR = 1.56, 95% CI: 1.04, 2.09). Taxi drivers attending nightclubs were six times (AOR = 6.04, 95% CI 1.97, 18.55) and watching pornography were four times (AOR = 4.44, 95% CI: 2.14, 9.19) more likely to engage in risky sexual practices respectively (see Table 4).

**Table 4:** Logistic regression of factors associated with risky sexual practices among taxi drivers in the Finoteselam town, northwest Ethiopia, 2023.

Variables		Frequency (%)		COR (95% CI)	AOR (95% CI)	*p*-value
		Risky	Non-risky			
Age	18–24 years	10 (12.5%)	70 (87.5%)	0.96 (0.37, 2.54)	1.94 (0.609,6.21)	0.26
	25–34 years	99 (47.4%)	110 (52.6%)	6.1 (2.88, 12.92)	6.4 (0.81, 9.51)	0.07
	≥35 years	9 (12.9%)	61 (87.1%)	1	1	
Residence	Urban	107 (34.3%)	205 (65.7%)	1.7 (0.84,2.49)	1.5 (0.576,4.22)	0.3
	Rural	11 (23.4%)	36 (76.6%)	1	1	
Educational status	1^ry^ school	12 (44.4%)	15 (55.6%)	2.02 (0.87, 4.69)	3.11 (0.88, 11.0)	0.07
	2ry school	66 (34.6%)	125 (65.4%)	1.33 (0.83, 2.14)	1.58 (0.80, 3.12)	0.1
	College	40 (28.4%)	101 (71.6%)	1	1	
Living status	Alone	84 (52.5%)	76 (47.5%)	5.36 (3.31,8.688)	3.47 (1.86, 6.48)	**0**.000**
	With family^a^	34 (17.1%)	165 (82.9%)	1	1	
Ownership	Owner of taxi	91 (35.1%)	168 (64.9%)	1.46 (0.88, 2.44)	2.08 (1.01, 4.25)	**0**.045*
	Non owner	27 (27%)	73 (73%)	1	1	
peers who started sex	Yes	46 (28.4%)	116 (71.6%)	0.68 (0.44,1.07)	0.64 (0.33,1.245)	0.19
	No	72 (36.5%)	125 (63.5%)	1	1	
Ever had STDs	Yes	15 (57.7%)	11 (42.3%)	1.9 (0.84, 4.35)	0.85 (0.28, 2.59)	0.7
	No	103 (41.5%)	145 (58.5%)	1	1	
Perceived risk STDs	No	33 (38.4%)	53 (61.6%)	1.37 (0.83, 2.28)	0.92 (0.44, 1.92)	0.8
	Yes	85 (31.1%)	188 (68.9%)	1	1	
SRH discussion	No	60 (47.6%)	66 (52.4%)	2.74 (1.73, 4.33)	2.08 (1.08, 4.00)	**0**.028*
	Yes	58 (24.9%)	175 (75.1%)	1	1	
substance use	Yes	73 (38.4%)	117 (61.6%)	1.71 (1.09, 2.69)	1.56 (1.04, 2.09)	**0**.047*
	No	45 (26.6%)	124 (73.4%)	1	1	
Attending night clubs	Yes	34 (79.1%)	9 (20.9%)	10.4 (4.8, 22.67)	6.04 (1.97, 18.55)	**0**.002*
	No	84 (26.6%)	232 (73.4%)	1	1	
Watching pornography	Yes	58 (65.2%)	31 (34.8%)	6.55(3.8, 11.03)	4.44(2.14, 9.19)	**0**.000**
	No	60(22.2%)	210(77.8%)	1	1	

^a^ parents and their sexual partners, SRH- sexual and reproductive health.

* *P* < 0.05.

** *P* < 0.001.

## Discussion

This study determined the higher prevalence of risky sexual practices among taxi drivers in northwest Ethiopia. The finding is consistent with studies in Ethiopia (27, 30), Thailand (25) and Bangladesh (47). The findings demonstrated that taxi drivers were extremely vulnerable to risky sexual practices (18). The study showed a high proportion of premarital sex among taxi drivers. This figure is comparable with findings from studies conducted in Ethiopia (30), Nepal (22), Bangladesh (47), and Thailand (25). The habit of multiple sexual partners in the study area was higher. This finding is consistent with studies in Nepal (22), South Africa (23), and low- and middle-income countries (25). The study found that a higher proportion of taxi drivers who did not intend to use during their contact with extra partners. The finding is comparable to studies conducted in Nepal (22), South Africa (23) and Ethiopia (27, 30).

This study revealed that living alone was associated with a three-fold probability of engaging in risky sexual practices than living with families (parents and spouse). This finding is consistent with studies in Bangladesh (47) and Thailand (25). This could be due to the fact that living alone provides taxi drivers with more opportunities to entertain themselves in nightclubs and unbridled freedom to have several sexual partners (27). Compared to taxi drivers who operate taxi under the supervision of another person who owns the vehicle, those who operate their own taxis are significantly more likely to engage in risky sexual practices, as demonstrated in the study. This could be due to the opportunity to experiment and participate in activities that are catalysts for risky sexual practices being greater for taxi drivers who own their own vehicles.

This study also showed that there was a greater than twofold greater opportunity for risky sexual practices among taxi drivers who did not discuss SRH with their families. This finding is comparable to studies conducted in the USA (48) and Ethiopia (49). However, a study in Ghana demonstrated that family communication does not affect an individual's sexual activity (35). This could be due to lack of perceptions and awareness of the detrimental health consequence of risky sexual practices and a misunderstanding of the importance of condom use.

The current study revealed that substance use, smoking, and illicit drug use were shown to be strong risk factors for risky sexual practices. Therefore, taxi drivers who had a habit of using substances were more likely to engage in risky sexual practices than those who did not. This finding is comparable to studies conducted in Bangladesh (47), Thailand (25), Poland (28), South Africa (29), and Ethiopia (30). This may be due to the fact that substance use can adversely affect adolescents' mental decision-making ability and conscious judgement (50). This could also be due to the fact that substance use creates a favourable opportunity (multiple sexual partners, attending night clubs, and watching pornography) to improve risky sexual practices of the individuals (51). Furthermore, the study demonstrated a significant correlation between nightclub attendance and risky sexual practices among taxi drivers in the study area. Taxi drivers who had experience attending nightclubs were six times more likely to engage in risky sexual practices than their counter participants. The figure is comparable to the study in USA (41). This may be due to the fact that taxi drivers attending nightclubs are more likely to have sexual relationships with commercial sexual workers, and substance use in the club may result in unlucky condom use and unwanted sexual relationships (25, 28, 29).

Similarly, the study showed that the odds of risky sexual practices among taxi drivers who had a habit of watching pornographic materials were four times higher than those who did not watch. This figure is comparable to studies in the US (38), New Works (39), and Ethiopia (40). This could be due to watching pornography drive taxi drivers for premarital sex, increased sexual desire, and motivation, which leads them to risky sexual practices (38).

### Limitations of the study

Recruitment of taxi drivers from those who were formally joined legal taxi association may miss those taxi drivers who did not join the taxi association formally as a result the study findings may not be representative of the entire population of taxi drivers and underestimation of the proportion of risky sexual practices. The use of a small number of female taxi drivers or taxi drivers in the study may compromise the general representativeness of the study findings for the various female taxi drivers.

Relying solely on self-reported data may compromise the actual study finding because taxi drivers might underreport their risky sexual practices. Reporting sexual activity is a sensitive issue and is considered taboo in the study community, so our results might not accurately reflect the exact sexual practices of general populations. The study findings may not be directly applicable to diverse populations of taxi drivers because sexual practices vary significantly between cultures and over time. Furthermore, the study findings do not provide information about taxi drivers' knowledge and awareness level about risky sexual factors and determinants factors.

## Conclusions and recommendations

This study highlights a concerning prevalence of risky sexual practices among taxi drivers in Finoteselam town, Northwest Ethiopia. Factors such as living alone, taxi ownership, neglect of sexual and reproductive health (SRH) discussions, substance use, exposure to pornography, and attending nightclubs were significantly associated variables with risky sexual practices.

Addressing this issue requires a multipronged approach. The Ministry of Health should implement national policies promoting sexual health and STD prevention, focusing on taxi drivers. Local health departments should provide accessible services such as STI/STD screening, counseling, and condom distribution, especially in areas frequented by taxi drivers. They should also encourage open SRH discussions and raise awareness about the harmful consequences of risky sexual practices. Transportation authorities should incorporate sexual health education into driver training and licensing requirements, while promoting safer sexual practices within the industry.

Taxi driver associations should advocate for their members' sexual health needs and act as intermediaries between drivers and health authorities. Media and communication channels should provide information and connect drivers with sexual health messages. Finally, researchers should conduct more studies, including longitudinal and qualitative research, to understand the social and environmental factors that contribute to risky sexual practices, focusing on larger sample sizes and including more female taxi drivers.

By working together, stakeholders can effectively address the issue of risky sexual practices among taxi drivers and promote safer sexual practices within this community.

## Data Availability

The original contributions presented in the study are included in the article/Supplementary Material, further inquiries can be directed to the corresponding author.
